# Oxidative stress is associated with suspected non‐alcoholic fatty liver disease and all‐cause mortality in the general population

**DOI:** 10.1111/liv.14562

**Published:** 2020-06-28

**Authors:** Turtushikh Damba, Arno R. Bourgonje, Amaal E. Abdulle, Andreas Pasch, Svenja Sydor, Eline H. van den Berg, Ron T. Gansevoort, Stephan J. L. Bakker, Hans Blokzijl, Robin P. F. Dullaart, Harry van Goor, Han Moshage

**Affiliations:** ^1^ Department of Gastroenterology and Hepatology University Medical Center Groningen University of Groningen Groningen the Netherlands; ^2^ School of Pharmacy Mongolian National University of Medical Sciences Ulaanbaatar Mongolia; ^3^ Department of Internal Medicine Division Vascular Medicine University Medical Center Groningen University of Groningen Groningen the Netherlands; ^4^ Institute for Physiology and Pathophysiology Johannes Kepler University Linz Linz Austria; ^5^ Department of Gastroenterology, Hepatology, and Infectious Diseases Otto von Guericke University Hospital Magdeburg Magdeburg Germany; ^6^ Department of Internal Medicine Division Nephrology University Medical Center Groningen University of Groningen Groningen the Netherlands; ^7^ Department of Endocrinology University Medical Center Groningen University of Groningen Groningen the Netherlands; ^8^ Department of Pathology and Medical Biology University Medical Center Groningen University of Groningen Groningen the Netherlands; ^9^ Department of Laboratory Medicine University Medical Center Groningen University of Groningen Groningen the Netherlands

**Keywords:** fatty liver index FLI, free thiols, NAFLD, oxidative stress

## Abstract

**Background & Aims:**

Non‐alcoholic fatty liver disease (NAFLD) is characterized by excessive lipid accumulation, inflammation and an imbalanced redox homeostasis. We hypothesized that systemic free thiol levels, as a proxy of systemic oxidative stress, are associated with NAFLD.

**Methods:**

Protein‐adjusted serum free thiol concentrations were determined in participants from the Prevention of Renal and Vascular End‐Stage Disease (PREVEND) cohort study (n = 5562). Suspected NAFLD was defined by the Fatty Liver Index (FLI ≥ 60) and Hepatic Steatosis Index (HSI > 36).

**Results:**

Protein‐adjusted serum free thiols were significantly reduced in subjects with FLI ≥ 60 (n = 1651). In multivariable logistic regression analyses, protein‐adjusted serum free thiols were associated with NAFLD (FLI ≥ 60) (OR per doubling of concentration: 0.78 [95% CI 0.64‐0.96], *P* = .016) even when adjusted for potential confounding factors, including systolic blood pressure, diabetes, current smoking, use of alcohol and total cholesterol (OR 0.80 [95% CI 0.65‐0.99], *P* = .04). This association lost its significance (OR 0.94 [95% CI 0.73‐1.21], *P* = .65) after additional adjustment for high‐sensitive C‐reactive protein. Stratified analyses showed significantly differential associations of protein‐adjusted serum free thiol concentrations with suspected NAFLD for gender (*P* < .02), hypertension (*P* < .001) and hypercholesterolemia (*P* < .003). Longitudinally, protein‐adjusted serum free thiols were significantly associated with the risk of all‐cause mortality in subjects with NAFLD (FLI ≥ 60) (HR 0.27 [95% CI 0.17‐0.45], *P* < .001).

**Conclusion:**

Protein‐adjusted serum free thiol levels are reduced and significantly associated with all‐cause mortality in subjects with suspected NAFLD. Quantification of free thiols may be a promising, minimally invasive strategy to improve detection of NAFLD and associated risk of all‐cause mortality in the general population.

**Abbreviations**
ALT: alanine aminotransferase
AST: aspartate aminotransferase
BMI: body mass index
CVD: cardiovascular disease
FLI: fatty liver index
GGT: gamma‐glutamyl‐transferase
HCC: hepatocellular carcinoma
HIS: Hepatic Steatosis Index
hs‐CRP: high‐sensitive C‐reactive protein
IBD: inflammatory bowel disease
NAFLD: non‐alcoholic fatty liver disease
NASH: non‐alcoholic steatohepatitis
PREVEND: Prevention of REnal and Vascular ENd‐Stage Disease
RNS: reactive nitrogen species
ROS: reactive oxygen species
RSS: reactive sulphur species
SAAs: sulphur‐based amino acids
T2D: type 2 diabetes
TG: triglycerides

**Key Points**

- Protein‐adjusted serum free thiol levels are reduced and significantly associated with all‐cause mortality in subjects with suspected Non‐Alcoholic Fatty Liver Disease (NAFLD) (FLI ≥ 60).
- Quantification of systemic free thiols may be a promising, minimally invasive strategy to improve detection of NAFLD and associated risk of all‐cause mortality in the general population.

## 1 INTRODUCTION

Non‐alcoholic fatty liver disease (NAFLD) is defined as an abnormal accumulation of triglycerides (TG) in hepatocytes in the absence of excessive alcohol consumption. NAFLD is emerging as the most prevalent chronic liver disease in Western countries. NAFLD encompasses a spectrum of diseases that ranges from simple steatosis to non‐alcoholic steatohepatitis (NASH), in combination with fibrosis. NASH can subsequently lead to cirrhosis with its known complications, such as hepatocellular carcinoma (HCC).^1^ Many co‐morbidities coincide with the development of NAFLD, such as obesity, insulin resistance and metabolic syndrome, including type 2 diabetes (T2D).^2^, ^3^ In the general population, suspected NAFLD can be estimated by calculating proxies of the disease, including the Fatty Liver Index (FLI) or the Hepatic Steatosis Index (HSI). Both of these scoring systems are considered to be potential predictors for NAFLD and are based on prominent risk factors, including obesity indices, plasma triglycerides, gamma‐glutamyl‐transferase (GGT), body mass index (BMI) and liver transaminases.^4^, ^5^

A number of previous studies demonstrated that inflammation significantly contributes to the progression of NAFLD. During NAFLD, hepatocytes no longer tolerate the toxicity of accumulated fatty acids, resulting in dysfunction of cellular homeostasis, including mitochondrial β‐oxidation and endoplasmic reticulum stress. Following this, an overproduction of endogenous reactive species (consisting of reactive oxygen species [ROS], reactive nitrogen species [RNS] and reactive sulphur species [RSS]) as well as an inflammatory signalling cascade in the liver is being generated.^6^, ^7^ An increased production of reactive species subsequently leads to hepatocellular injury, which in turn results in secretion of inflammatory cytokines (TNF‐α, IL‐6, IL‐10) and cellular death. The pro‐inflammatory signalling pathways, increased β‐oxidation in mitochondria and peroxisomes involved in this process lead to dysregulation of antioxidant homeostasis.^8^

Thiols (R‐SH) comprise a group of organosulphur compounds that can be found mainly in proteins (e.g. albumin) that contain sulphur‐based amino acids (SAAs) as well as in low‐molecular‐weight (LMW) molecules like cysteine, homocysteine and glutathione. Thiols are known to be involved in various biological processes, such as enzymatic catalysis, cell signalling and metal complexing in the body.^9^ Most importantly, plasma or serum thiols are considered as a global marker of the systemic load of reactive species and as potent anti‐oxidants because of their high reducing activity.^10^ According to recently proposed terminology, reactive species can be identified as ROS, as well as RNS and RSS, which are collectively referred to as the ‘Reactive Species Interactome’ (RSI).^9^ Depending on their redox state, thiols are classified as reduced or “free” thiols (R‐SH) and oxidized or “bound” thiols, in which case a thiol is bound to another thiol via a disulfide bridge (R‐SS‐R’). In the circulation, the largest share of free thiols is embedded within the single cysteine residue (Cys^34^) of albumin (HSA‐SH) which exerts its antioxidant capacity. Remaining free thiols are classified as LMW free thiols, and the sum of protein free thiols and LMW free thiols is defined as *total free thiols*. Free thiols are able to scavenge reactive species and form disulphide bonds. Generally, total free thiol levels in serum could be interpreted as a direct and reliable reflection of the systemic redox system since they are readily oxidized by reactive species.^11^, ^12^ Typically, high concentrations of serum free thiols are representative of a more beneficial or ‘healthy’ redox status. Changes in serum free thiol levels have been reported for many risk factors in which reactive species are known to play a prominent role, such as ageing, smoking, alcohol consumption, as well as for several diseases including inflammatory bowel disease (IBD), cardiovascular disease (CVD), obesity and ischaemia‐reperfusion injury.^13^, ^14^, ^15^ Only one study reported that total serum thiol concentration is reduced while thiol‐disulphide level is increased in NASH patients, compared to healthy controls.^16^

In this study, we determined systemic levels of serum free thiols in 5562 participants included in the Prevention of Renal and Vascular End‐stage Disease (PREVEND) cohort, a large population‐based cohort study from the Northern part of the Netherlands. Firstly, we compared protein‐adjusted serum free thiol levels between subjects with FLI < 60 and FLI ≥ 60 values and established associations between free thiol levels and multiple clinical, biochemical and NAFLD‐specific parameters. Secondly, we investigated the association between baseline protein‐adjusted serum free thiol concentrations and the risk of all‐cause mortality during a follow‐up of 10 years.

## 2 MATERIALS AND METHODS

### 2.1 Study population

This study used data from the Prevention of REnal and Vascular ENd‐stage Disease (PREVEND) cohort study.^17^ This is a large, prospective population‐based cohort study with participants from the Northern part of the Netherlands. The PREVEND study was set up to investigate cardiovascular and renal disease outcomes. From 1997 to 1998, 85 421 inhabitants aged 28‐75 years from the Northern part of the Netherlands, received a questionnaire asking information about demographics, medication use, cardiovascular disease and pregnancy, including a request to supply an early morning urine sample. Participants who had a previous diagnosis of type 1 diabetes mellitus, insulin‐treated type 2 diabetes mellitus and pregnant women were excluded from the study. In total, 40 856 subjects responded to the questionnaire and were analyzed for urinary albumin concentrations. Subjects with a urinary albumin concentrations ≥10 mg/L (n = 6000) were invited to visit the outpatient research clinic, as well as a random selection of participants with urinary albumin concentrations <10 mg/L (n = 2592). The PREVEND study consisted of a total of 8592 participants who completed the full study program.^18^ However, for the current study we excluded subjects (n = 3030) of which data on serum levels of free thiols and clinical and biochemical variables to calculate the Fatty Liver Index (FLI), as a proxy of NAFLD, were not available. This study was approved by the Institutional Review Board (IRB) of the University Medical Center Groningen (UMCG). The study was conducted in accordance with the principles of the Declaration of Helsinki (2013). All study participants provided written informed consent.

### 2.2 Data collection

All study participants visited the outpatient research clinic of the UMCG, Groningen, the Netherlands. During the first visit, participants were requested to complete a questionnaire that contained information about demographics, health status, history of cardiovascular diseases (CVD), use of medications and lifestyle (e.g. self‐reported smoking and alcohol consumption). Smoking was categorized as either current smoking or never or previous smoking. Alcohol consumption was documented with the assumption of one alcoholic drink to contain 10 grams of alcohol. History of cardiovascular disease included the following: hospitalization for myocardial ischaemia, obstructive coronary artery disease or revascularization procedures. Subsequently, anthropometric measurements were performed, including height (meters), weight (kilograms), body‐mass index (BMI, weight divided by squared height), waist circumference (cm, defined as the smallest girth between rib cage and iliac crest), and waist/hip ratio (waist circumference divided by the largest girth between waist and thigh).^19^, ^20^ During the second visit, systolic and diastolic blood pressure was measured automatically every minute until 8 minutes in supine position (Dinamap XL Model 9300 series device, Johnson & Johnson Medical). Blood pressure was defined as the average of the last two measurements in this procedure. Next, venous serum samples were withdrawn after an overnight fast while the participants had rested for 15 minutes. In addition, patients were asked to collect 24‐hours urine specimens after they were provided with both oral and written instructions. In the current study, data were used of participants who completed the second screening evaluation in the PREVEND study.

### 2.3 Laboratory measurements

Urinary albumin excretion (UAE) and high‐sensitive C‐reactive protein (hs‐CRP) were measured by nephelometry (Dade Behring Diagnostics). UAE was measured twice in two different 24‐ hours urine specimens and the average of these was used in further analyses. Serum total cholesterol and serum glucose levels were measured by dry chemistry (Eastman Kodak). Low‐density lipoprotein (LDL) cholesterol was determined by the Friedewald formula (if triglycerides ≤4.5 mmol/L). High‐density lipoprotein (HDL) cholesterol was measured using a homogeneous method (direct HDL, AerosetTM System, Abbott Laboratories). Triglycerides were measured using an enzymatic method. Serum creatinine was measured with an enzymatic method as well (Roche Modular, Roche Diagnostics). Serum cystatin C was measured using the Gentian Cystatin C Immunoassay (Gentian AS) on a modular analyzer (Roche Diagnostics). Cystatin C was directly calibrated using a standard from the manufacturer (according to the International Federation of Clinical Chemistry Working Group for Standardization of Serum Cystatin C).^21^ Serum ALT and AST were measured using the standardized kinetic method with pyridoxal phosphate activation (Roche Modular P, Roche Diagnostics). Serum GGT was assayed by an enzymatic colorimetric method (Roche Modular P, Roche Diagnostics).

### 2.4 Measurement of serum free thiols

Serum samples were stored at −80°C until analysis to avoid significant alterations in free thiol stability. Serum free thiol concentrations were measured as previously described, with minor modifications.^22^, ^23^ After thawing, serum samples were diluted four‐fold using 0.1 mol/L Tris buffer (pH 8.2). Using the Varioskan microplate reader (Thermo Scientific, Breda, the Netherlands), background absorption was measured at 412 nm, together with a reference measurement at 630 nm. Subsequently, 20 μL 1.9 mmol/L 5,5′‐dithio‐bis(2‐nitrobenzoic acid) (DTNB, Ellman's Reagent, CAS‐number 69‐78‐3, Sigma Aldrich Corporation) in 0.1 mol/L phosphate buffer (pH 7.0) was added to the samples and absorbance was measured again after the samples were incubated for 20 min at room temperature. Final concentrations of serum free thiols were established by parallel measurement of an L‐cysteine (CAS‐number 52‐90‐4, Fluka Biochemika) calibration curve (concentration range from 15.625 to 1000 μmol/L) in 0.1 mol/L Tris/10 mmol/L EDTA (pH 8.2). Intra‐ and interday coefficients of variation (CV) of all measurement values were below 10%. Ultimately, serum free thiol concentrations were adjusted to total serum protein levels (measured according to standard procedures) by calculating the free thiol/total protein ratio (μmol/g of protein). This adjustment was performed since serum proteins harbour the largest amount of free thiols and therefore largely determine the amount of potentially detectable free thiols.^24^

### 2.5 Study outcomes and definitions

The estimated glomerular filtration rate (eGFR) was calculated using the combined creatinine cystatin C‐based Chronic Kidney Disease Epidemiology Collaboration (CKD‐EPI) equation.^25^ Type 2 diabetes (T2D) was defined as a fasting glucose level ≥7.0 mmol/L, a random glucose level ≥11.1 mmol/L, self‐report of a physician's diagnosis or the use of antidiabetic medications according to the guidelines of the American Diabetic Association (ADA). The algorithm of the Fatty Liver Index (FLI) was used as a proxy for the diagnosis of suspected NAFLD.^5^ The FLI was calculated according to the following formula: FLI = [e (0.953 × loge (triglycerides) + 0.139 × BMI + 0.718 × loge (GGT) + 0.053 × waist circumference − 15.745)]/[1 + e (0.953 × loge (triglycerides) + 0.139 × BMI + 0.718 × loge (GGT) + 0.053 × waist circumference − 15.745)] × 100. The optimal cut‐off value of the FLI for detecting NAFLD is established as 60 with a corresponding sensitivity of 61%, specificity of 86% and an accuracy of 84% as determined by ultrasonography.^5^ Therefore, FLI ≥ 60 was used as a definition of suspected NAFLD, which is used nowadays as one of the best‐validated steatosis scores for large scale screening studies.^26^ Alternatively, we used the Hepatic Steatosis Index (HSI), which has been used previously in predominantly Asian populations and is defined as follows^4^: HSI = 8 × ALT/AST ratio + BMI (+2, if diabetes; +2, if female). The optimal cut‐off value of the HSI for detecting NAFLD is a score of 36. In the above equations, BMI was expressed as kg/m^2^, triglycerides as mmol/L and gamma‐glutamyltransferase (GGT), alanine aminotransferase (ALT) and aspartate aminotransferase (AST) as U/L.

Metabolic syndrome (MetS) was defined according to the revised National Cholesterol Education Program Adult Treatment Panel (NCEP‐ATP) III criteria. Study participants were assigned to have MetS when at least three of the following five criteria were fulfilled: (a) waist circumference >102 cm for men and >88 cm for women; (b) plasma triglycerides ≥1.7 mmol/L; (c) HDL cholesterol <1.0 mmol/L for men and <1.3 mmol/L for women; (d) hypertension (blood pressure ≥130/85 mmHg or the use of antihypertensive drugs); (e) hyperglycemia (fasting glucose ≥5.6 mmol/L or the use of glucose lowering drugs).

Information on death (all‐cause mortality) was obtained from the Dutch national registry of all hospital discharge diagnoses (Prismant). This information was classified in accordance with the International Statistical Classification of Diseases (ICD‐10) and the International Classification of Health Interventions.^27^

### 2.6 Statistical analyses

Demographic, clinical and biochemical characteristics of the study population were presented as means ± standard deviations (SD), proportions *n* with corresponding percentages (%) or medians [interquartile range (IQR)] in case of non‐normal distributions. Assessment of normality was performed using histograms and normal probability plots (Q‐Q plots). Between‐group comparisons were performed using independent sample *t*‐tests or Mann‐Whitney *U*‐tests in case of continuous variables, while chi‐square tests were used in case of nominal variables. Protein‐adjusted serum free thiol concentrations were ^2^log‐transformed prior to analysis to facilitate interpretation of the results (expressed as per doubling). Univariable and multivariable logistic regression analyses were performed to evaluate associations between serum free thiol concentrations and NAFLD parameters expressed as odds ratios (OR) (per doubling) with corresponding 95% confidence intervals (CI). Stratified analyses were performed to examine the association between serum free thiols and NAFLD across various subgroups. Survival distributions for subjects with and without NAFLD were assessed according to tertiles of protein‐adjusted serum free thiol concentrations using Kaplan‐Meier curves and compared to each other using log‐rank tests. Survival time was defined from baseline (time of serum sample withdrawal) until the date of the last examination participants attended, either death or January 1, 2010 (end of follow‐up period). Subsequently, Cox proportional hazards regression analyses were performed to assess associations between protein‐adjusted serum free thiol concentrations and the risk of all‐cause mortality, expressed as hazard ratios (HRs) (per doubling) with corresponding 95% CIs. Univariable associations were followed by multivariable models to adjust for potential confounding factors. Data analysis was performed using SPSS Statistics 25.0 software package (SPSS Inc) and data visualization using GraphPad Prism 5.0 (GraphPad software). Two‐tailed *P*‐values ≤.05 were considered statistically significant.

## 3 RESULTS

### 3.1 Baseline characteristics of the study population

Baseline characteristics of the study population are presented in Table 1. The study population consisted of 5562 participants, of whom 1651 (29.7%) subjects were classified with a FLI ≥ 60. Participants classified with a FLI ≥ 60 were significantly older, as compared to subjects with a FLI < 60 (56.0 years vs 49.8 years, *P* < .001). In addition, subjects with a FLI ≥ 60 more frequently had a history of cardiovascular disease (*P* < .001), MetS (*P* < .001) and more often used antihypertensive medication (*P* < .001) and lipid‐lowering drugs (*P* < .001). Moreover, anthropometric tests (ie BMI, waist circumference, waist/hip ratio), cholesterol levels and liver transaminase levels were higher in subjects with a FLI ≥ 60 (*P* < .001 for all). Conversely, LDL‐cholesterol levels were not found to be significantly different between groups. With regard to serum levels of protein‐adjusted free thiols, we observed significantly reduced concentrations in subjects with a FLI ≥ 60, as compared to subjects with a FLI < 60 (4.91 μmol/L/g vs 5.05 μmol/L/g, *P* < .001).

**TABLE 1:** Clinical and laboratory characteristics including protein‐adjusted serum free thiols in 3911 subjects with a fatty liver index (FLI) < 60 and 1651 subjects with a FLI ≥ 60

	FLI < 60 n = 3911	FLI ≥ 60 n = 1651	*P*‐value
Age year, median (IQR)	49.84 (42.11‐59.43)	55.99 (47.96‐65.78)	**<.001**
Gender (men), n (%)	1599 (40.9)	1096 (66.4)	**<.001**
Ethnicity			
Caucasian, n (%)	3727 (95.3)	1575 (95.4)	.176
Asian, n (%)	85 (2.2)	26 (1.6)	
Black, n (%)	31 (0.8)	21 (1.3)	
Other, n (%)	41 (1.0)	17 (1.0)	
Unknown, n (%)	27 (0.7)	12 (0.7)	
Current smokers, n (%)	1093 (28.3)	440 (26.9)	.312
Use of alcohol, n (%)	2975 (76.7)	1190 (72.5)	**<.001**
BMI (kg/m^2^), median (IQR)	24.62 (22.83‐26.71)	30.15 (25.02‐32.91)	**<.001**
Waist circumference (cm), median (IQR)	86 (79‐93)	104 (99‐110)	**<.001**
Waist/hip ratio, mean ± SD	0.86 ± 0.07	0.96 ± 0.07	**<.001**
Systolic blood pressure (mm Hg), median (IQR)	120 (110‐133)	135 (123‐147)	**<.001**
Diastolic blood pressure (mm Hg), median (IQR)	71 (65‐77)	78 (71‐84)	**<.001**
Antihypertensive medication, n (%)	471 (12.4)	485 (30.1)	**<.001**
Lipid‐lowering drugs, n (%)	171 (4.5)	161 (10)	**<.001**
History of cardiovascular disease, n (%)	97 (2.5)	87 (5.3)	**<.001**
MetS, n (%)	309 (7.9)	958 (58.1)	**<.001**
Glucose (mmol/L), median (IQR)	4.70 (4.40‐5.10)	5.10 (4.60‐5.60)	**<.001**
Insulin (mU/L), median (IQR)	6.80 (5.10‐9.20)	12.70 (9.40‐18.40)	**<.001**
HOMA‐IR (mU × mmol/L^2^/22.5), median (IQR)	1.43 (1.03‐2.00)	2.88 (2.04‐4.35)	**<.001**
HOMA‐β (%), median (IQR)	25.38 (18.31‐35.13)	46.15 (33.02‐66.64)	**<.001**
Urinary albumin excretion (mg/24 h), median (IQR)	7.78 (5.76‐12.55)	11.03 (7.08‐23.47)	**<.001**
eGFR (mL/min/1.73m^2^), median (IQR)	96.30 (84.86‐106.07)	89.20 (77.26‐100.63)	**<.001**
hs‐CRP (mg/L), median (IQR)	1.01 (0.48‐2.27)	2.36 (1.17‐4.22)	**<.001**
ALT (U/L), median (IQR)	15 (12‐20)	23 (17‐32)	**<.001**
AST (U/L), median (IQR)	21 (19‐25)	25 (21‐30)	**<.001**
ALP (U/L), median (IQR)	59 (49‐71)	58 (57‐79)	**<.001**
GGT (U/L), median (IQR)	19 (14‐27)	41 (29‐62)	**<.001**
Total cholesterol (mmol/L), mean ± SD	5.31 ± 0.99	5.78 ± 1.05	**<.001**
Non‐HDL cholesterol (mmol/L), median (IQR)	3.91 (3.30‐4.58)	4.64 (3.98‐5.36)	**<.001**
LDL cholesterol (mmol/L), median (IQR)	3.32 (2.70‐4.08)	3.48 (2.69‐4.22)	.446
HDL cholesterol (mmol/L), median (IQR)	1.30 (1.11‐1.51)	1.06 (0.9232‐1.23)	**<.001**
Triglycerides (mmol/L), median (IQR)	0.94 (0.71‐1.25)	1.72 (1.29‐2.33)	**<.001**
Free thiols (protein‐adjusted) (µmol/L/g), mean ± SD	5.05 ± 0.99	4.91 ± 1.02	**<.001**

Bold *P*‐values indicate statistical significance.

Data are presented as mean ± standard deviation (SD) for normally distributed data or median with interquartile ranges (IQR) for non‐normally distributed data.

Abbreviations: BMI, Body Mass Index; MetS, Metabolic syndrome; HOMA‐IR, Homeostatic Model Assessment of Insulin Resistance; HOMA‐β, Homeostatic Model Assessment of β cell function; hs‐CRP, high sensitive C reactive protein; ALT, Alanine Aminotransferase; AST, Aspartate aminotransferase; GGT, gamma‐glutamyltransferase; HDL, High‐Density Lipoprotein; LDL, Low‐Density Lipoprotein.

### 3.2 Associations between protein‐adjusted serum free thiol levels and FLI and HSI scores

Multivariable logistic regression analyses were subsequently performed in order to establish the extent to which serum levels of free thiols were associated with a FLI ≥ 60 (Table 2). In the age‐ and gender‐adjusted analysis, we found a significant association between protein‐adjusted free thiols (^2^log‐transformed, per doubling of concentration) and FLI (*Model 2*: OR 0.78 [95% CI 0.64‐0.96], *P* = .016). This association remained statistically significant after additional adjustment for systolic blood pressure, diabetes, current smoking, use of alcohol and total cholesterol (*Model 3*: OR 0.80 [95% CI 0.65‐0.99], *P* = .04). After additional adjustment for hs‐CRP, this association lost significance (*Model 4:* OR 0.94 [95% CI 0.73‐1.21], *P* = .65). Similar results were observed in the analysis for HSI (Table S1). For instance, the association between HSI and serum levels of protein‐adjusted free thiols (^2^log‐transformed, per doubling of concentration) only lost its significance after additional adjustment for hs‐CRP (OR 0.87 [95% CI 0.68‐1.10], *P* = .24). Stratified analyses for the association between protein‐adjusted serum free thiols (per doubling) and FLI scores are presented in Table 3. Stratification by gender, the presence of hypertension and the presence of hypercholesterolemia showed significant differences between groups. Corresponding HRs were lower for female subjects (*P*_interaction_ = 0.02), subjects without hypertension (*P*_interaction_ = 0.001) and subjects without hypercholesterolemia (*P*_interaction_ = 0.003). Comparable results were obtained in stratified analyses when using the HSI instead of the FLI (Table S2).

**TABLE 2:** Multivariable logistic regression analysis to test the relationship between FLI and serum levels of protein‐adjusted serum free thiols (^2^log‐transformed)

	Model 1		Model 2		Model 3		Model 4	
	OR [95% CI]	*P*‐value	OR [95% CI]	*P*‐value	OR [95% CI]	*P*‐value	OR [95% CI]	*P*‐value
Free thiols (^2^log)	0.65 [0.54‐0.78]	<.001	0.78 [0.64‐0.96]	.016	0.80 [0.65‐0.99]	.04	0.94 [0.73‐1.21]	.65
Age			1.03 [1.03‐1.04]	<.001	1.00 [0.99‐1.01]	.26	1.00 [0.99‐1.01]	.62
Gender (reference = male)			0.35 [0.31‐0.40]	<.001	0.35 [0.31‐0.40]	<.001	0.33 [0.28‐0.39]	<.001
Diabetes (no = reference)					3.92 [2.63‐5.82]	<.001	4.02 [2.35‐6.90]	<.001
Current smoking (reference = no)					1.01 [0.88‐0.1.17]	.87	0.98 [0.83‐1.16]	.82
Use of alcohol (reference = no)					0.69 [0.59‐0.80]	<.001	0.69 [0.58‐0.82]	<.001
Systolic blood pressure					1.03 [1.03‐1.04]	<.001	1.03 [1.03‐1.04]	<.001
Total cholesterol					1.54 [1.44‐1.64]	<.001	1.54 [1.44‐1.66]	<.001
hs‐CRP							1.08 [1.06‐1.10]	<.001

Note

Model 1: crude.

Model 2: model 1 + additional correction for age and gender.

Model 3: model 2 + additional correction for systolic blood pressure, diabetes, current smoking, use of alcohol and total cholesterol.

Model 4: model 3 + additional correction for hs‐CRP.

**TABLE 3:** Stratified analyses for the association between ^2^log‐transformed protein‐adjusted serum free thiols and the fatty liver index (FLI) across various subgroups. Stratifications by gender, hypertension and hypercholesterolemia showed significant interactions

Variable	Total (n)	OR*	95% CI	*P*‐value (interaction)
Overall	5562	0.80	0.65‐0.99	**.042**
Gender				
Female	2815	0.63	0.46‐0.87	**.020**
Male	2639	0.96	0.72‐1.27	
BMI				
<25.0	2167	3.30	1.11‐9.79	.902
>25.0	3275	0.81	0.63‐1.05	
Albuminuria				
No	4813	0.83	0.66‐1.05	.060
Yes	639	0.86	0.51‐1.45	
Hypertension				
No	3765	0.78	0.59‐1.03	**.001**
Yes	1690	0.88	0.63‐1.22	
CVD history				
No	5273	0.83	0.66‐1.03	.200
Yes	181	0.42	0.15‐1.19	
Diabetes				
No	5322	0.82	0.66‐1.02	.386
Yes	132	0.62	0.21‐1.88	
Smoking				
No	3936	0.81	0.63‐1.04	.611
Yes	1518	0.77	0.52‐1.15	
Alcohol consumption				
No	1336	0.64	0.43‐0.96	.209
Yes	4118	0.88	0.68‐1.14	
Hypercholesterolemia				
No	3904	0.77	0.59‐1.00	**.003**
Yes	1579	0.86	0.60‐1.25	

Abbreviations: BMI, body‐mass index; CI, confidence interval; CV, cardiovascular; CVD, cardiovascular disease; OR, odds ratio.

* Adjusted for potential confounding factors (gender, age, history of diabetes, current smoking, alcohol consumption, blood pressure and hypercholesterolemia).

Bold *P*‐values indicate statistical significance.

### 3.3 Protein‐adjusted serum free thiols and risk of all‐cause mortality

**FIGURE 1:**
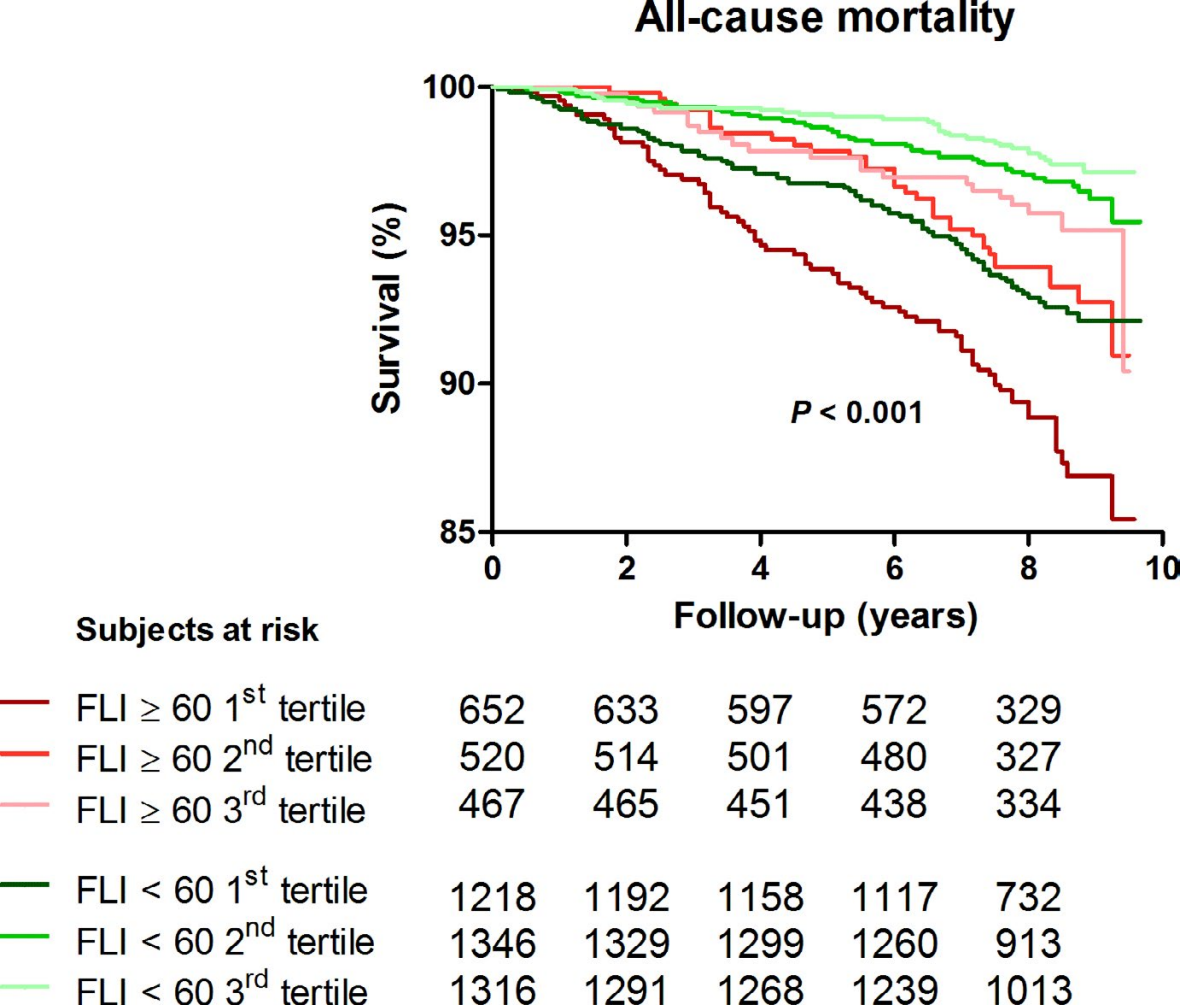
Kaplan‐Meier survival distributions for tertiles of protein‐adjusted serum free thiol concentrations (μmol/L/g). Kaplan‐Meier curve representing survival with the highest mortality rate occurring in the lowest tertile of protein‐adjusted serum free thiols in both groups (log‐rank test, *P* < .001)

During follow‐up, 291 (5.2%) subjects died (FLI < 60, n = 162 (4.1%), FLI ≥ 60, n = 129 (7.8%)). Kaplan‐Meier survival analysis showed a significantly differential survival distribution between tertiles of protein‐adjusted serum free thiols among subjects with a FLI < 60 and FLI ≥ 60 (Figure 1, *P* < .001, log‐rank test). Cox proportional hazards regression analyses showed a significant inverse predictive association between ^2^log‐transformed protein‐adjusted serum free thiol concentrations and the risk of all‐cause mortality for subjects with a FLI < 60 (Table 4A, model 1, HR per doubling of concentration 0.33 [0.22‐0.50], *P* < .001) and subjects with a FLI ≥ 60 (Table 4B, model 1, HR per doubling of concentration 0.27 [0.17‐0.45], *P* < .001). This association lost its significance after adjustment for potential confounders in subjects with a FLI < 60 (Table 4B, model 4, HR per doubling of concentration 0.78 [0.44‐1.39], *P* = .41), while it remained statistically significant in subjects with a FLI ≥ 60 (Table 4B, model 4, HR per doubling of concentration 0.50 [0.27‐0.95], *P* = .03). Similar results were obtained in Cox proportional hazard regression analyses using HSI instead of FLI, showing a statistically significant inverse association between ^2^log‐transformed protein‐adjusted serum free thiol concentrations and the risk of all‐cause mortality for subjects with both an HSI < 36 and HSI ≥ 36 (Table S3). However, statistical significance vanished after adjustment for potential confounders in subjects of both subgroups, with the exception of the highest tertile of protein‐adjusted serum free thiol concentrations in the group with HSI ≥ 36 (Table S3B, model 4, HR per doubling of concentration 0.39 [0.16‐0.94], *P* = .04).

**TABLE 4:** Cox proportional hazards regression models of the association between ^2^log‐transformed protein‐adjusted serum free thiols and potential confounding factors with all‐cause mortality, for patients with FLI < 60 (A) and FLI ≥ 60 (B)

	HR per doubling	Tertiles of protein‐adjusted serum free thiols		
		<4.65 μmol/g	4.65‐5.46 μmol/g	>5.46 μmol/g
(A) FLI < 60				
Model 1	0.33 [0.22‐0.50] ***P* < .001**	1.00 (Reference)	0.44 [0.31‐0.64] ***P* < .001**	0.33 [0.22‐0.49] ***P* < .001**
Model 2	0.75 [0.45‐1.24] *P* = .26	1.00 (Reference)	0.72 [0.50‐1.05] *P* = .09	0.72 [0.47‐1.11] *P* = .14
Model 3	0.77 [0.46‐1.27] *P* = .30	1.00 (Reference)	0.73 [0.50‐1.07] *P* = .11	0.71 [0.46‐1.09] *P* = .12
Model 4	0.78 [0.44‐1.39] *P* = .41	1.00 (Reference)	0.85 [0.57‐1.28] *P* = .44	0.68 [0.42‐1.11] *P* = .13
(B) FLI ≥ 60				
Model 1	0.27 [0.17‐0.45] ***P* < .001**	1.00 (Reference)	0.54 [0.36‐0.81] ***P* = .003**	0.37 [0.23‐0.60] ***P* < .001**
Model 2	0.62 [0.36‐1.06] *P* = .08	1.00 (Reference)	0.80 [0.53‐1.21] *P* = .29	0.66 [0.40‐1.09] *P* = .11
Model 3	0.65 [0.38‐1.12] *P* = .12	1.00 (Reference)	0.84 [0.55‐1.27] *P* = .41	0.69 [0.42‐1.15] *P* = .15
Model 4	0.50 [0.27‐0.95] ***P* = .03**	1.00 (Reference)	0.87 [0.55‐1.36] *P* = .53	0.64 [0.36‐1.14] *P* = .13

Note

Model 1: crude.

Model 2: model 1, age‐ and gender‐adjusted.

Model 3: model 2, adjusted for systolic blood pressure, diabetes, current smoking, use of alcohol and total cholesterol.

Model 4: model 3, additionally adjusted for hs‐CRP.

Bold *P*‐values indicate statistical significance.

Abbreviation: HR, hazard ratio.

## 4 DISCUSSION

In this study, we reported that protein‐adjusted serum free thiol concentrations, as a marker of the systemic redox status, were lowered in subjects with suspected NAFLD (FLI ≥ 60). In addition, protein‐adjusted serum free thiols were significantly associated with an increased risk of all‐cause mortality in subjects with suspected NAFLD in this population‐based cohort. Multivariable regression analyses showed maintenance of this significant association after adjustment for potential confounding factors, including the adjustment for systolic blood pressure, diabetes, current smoking, use of alcohol and total cholesterol in subjects with FLI ≥ 60. As expected, this association lost its significance after additional adjustment for high‐sensitive C‐reactive protein (hs‐CRP), indicating that inflammation and oxidative stress are both associated with NAFLD and not independent of each other.^28^ Stratified analyses showed that there were significantly differential associations of protein‐adjusted serum free thiol concentrations (per doubling) by gender, hypertension and hypercholesterolemia. Our results were further confirmed by comparable associations with the Hepatic Steatosis Index (HSI > 36), which is also a widely applied and recommended proxy to determine NAFLD in large population‐based cohort studies.^4^, ^5^ Taken together, the current study demonstrated that protein‐adjusted serum free thiols could be a prominent minimally invasive marker of reactive species‐driven development of NAFLD and are associated with the risk of all‐cause mortality in subjects with suspected NAFLD.

NAFLD, thought to be caused by an imbalanced influx of free fatty acids (FFAs) and excessive accumulation of triglycerides in hepatocytes, is strongly associated with insulin resistance and metabolic syndrome (MetS). During the development of NAFLD, FFA governing transcription regulators are disrupted (e.g. the transcription factors peroxisome proliferator‐activated receptor alpha [PPARα], or sterol regulatory element‐binding proteins [SREBPs]) causing inappropriate activation of pro‐inflammatory signalling pathways (via protein‐kinase B [AKT] or AMP‐activated protein kinase [AMPK]) that contribute to the production of pro‐inflammatory cytokines such as IL‐6, TNF‐α or IL‐1β and increased hepatocellular damage.^8^, ^29^ Concurrently, a shift in redox balance occurs through the combined sequence of mitochondrial dysfunction, impaired oxidation of free fatty acids (FFAs) and toxicity of excessively accumulated triglycerides. In our study, subjects with FLI ≥ 60 had a significantly higher frequency of previous cardiovascular disease and MetS as well as significantly increased plasma concentrations of triglycerides, alanine aminotransferase (ALT) and aspartate aminotransferase (AST), as compared to subjects without suspected NAFLD (FLI < 60). Most importantly, protein‐adjusted free thiol concentrations were significantly lower in subjects with FLI ≥ 60. These results were consistent in subjects having an HSI > 36. Altered serum thiol balance in NAFLD has been reported in only one study before. Asil *et al* reported that serum *total* thiols were reduced in patients with NASH and simple steatosis as compared to healthy controls (n = 90).^16^ In comparison to our data, that study focused on total/native thiol ratios, included relatively low numbers of patients and applied liver biopsy to define NAFLD. Several other studies reported that there were no significant differences with regard to total serum thiol concentrations in subjects with insulin resistance (IR), type 2 diabetes (T2D).^30^, ^31^, ^32^ Additionally, in paediatric subjects, increased serum thiols such as cysteine and homocysteine were observed in patients with NAFLD, while they were reduced in patients with NASH or liver fibrosis.^33^ However, these studies focused on thiol/disulphide homeostasis using different measurement protocols i.e. distinct thiol‐reactive reagents which compromises comparability of results between studies as measurements of either free thiols or total thiols lead to different classifications and terminology.^34^ In addition, all these studies were based on datasets with relatively low numbers of study participants or they focused on different types of populations, e.g. solely on paediatric or female subjects.

Oxidative stress is referred to as an imbalance between oxidant and anti‐oxidant substances. In NAFLD, the antioxidant system is disrupted because of excessive fat accumulation‐mediated endoplasmic reticulum (ER) stress and mitochondrial β‐oxidation dysfunction, leading to oxidative stress‐induced complications caused by endogenous production of reactive species.^6^, ^7^ It should be noted that serum free thiols have been considered a prominent antioxidant marker in serum because of their potent capacity to scavenge reactive species.^9^, ^14^, ^31^ High‐sensitive C‐reactive protein (hs‐CRP) has been reported to be a prominent ROS‐induced inflammatory marker in NAFLD.^35^ A diminished antioxidant capacity is significantly associated with hs‐CRP during disrupted redox homeostasis in multiple oxidative stress‐related diseases.^36^, ^37^, ^38^, ^39^ Similarly, in our study, serum hs‐CRP levels were significantly increased in subjects with FLI ≥ 60. In addition, in multivariable regression analyses, the persistent statistically significant associations of ^2^log‐transformed protein‐adjusted serum free thiols and systolic blood pressure, diabetes, current smoking, use of alcohol and total cholesterol with FLI ≥ 60 lost their significances after adjustment for hs‐CRP. The same results were obtained in the analysis of the HSI > 36 group. This similar association of hs‐CRP and thiols has been observed in several studies related to antioxidant homeostasis. For instance, one study found a negative correlation between hs‐CRP levels and thiol/disulphide ratio and a positive correlation with total thiols during acute appendicitis in children (n = 80).^40^ In addition, in patients with inflammatory bowel disease (IBD), hs‐CRP was also significantly inversely associated with free thiols.^14^, ^41^ These results further underscore that systemic free thiols are significantly associated with hs‐CRP as oxidative stress‐induced acute inflammation marker. Interestingly, in our stratified analyses, women with suspected NAFLD had a higher risk of impaired free thiol status. In agreement, Ates *et al* found that iron and the antioxidant enzyme ferroxidase activity was higher, while total plasma native thiol level was lower in women (n = 95) with obesity and insulin resistance (IR).^32^ Furthermore, our results support the fact that dysregulation of redox homeostasis is a crucial indicator in the presence of NAFLD.

In the future, free thiols could be further investigated for their potential to be implemented as a diagnostic or monitoring tool in NAFLD. Recent studies reported that systemic free thiol levels were significantly associated with heart failure, inflammatory bowel disease and levels of triglycerides and VLDL.^13^, ^14^, ^30^ Furthermore, dynamics of free thiols in serum could be a useful characteristic to determine the severity of disease. For instance, rapidly increased systemic free thiol levels were observed during the recovery phase of systemic sclerosis patients^42^ indicating that hypoxia elicits upregulation of the antioxidant status. In the present study, serum free thiol levels were significantly lowered in subjects with FLI ≥ 60 compared to FLI < 60. Since systemic free thiols (R‐SH) are considered to be amenable to therapeutic manipulation, it could also become a beneficial treatment target in NAFLD. In this regard, hydrogen sulphide (H_2_S) or precursors like N‐acetylcysteine (NAC) and glutathione as low molecular weight thiol‐containing compounds (and many other antioxidant supplementations) are considered to be potential treatment options to correct an imbalanced redox status in diseases like NAFLD.^43^, ^44^ Endogenous production of H_2_S is reduced in the cirrhotic liver, while exogenous H_2_S supplementation prevents NASH in an animal experimental model via abating oxidative stress and suppressing inflammation.^45^ In addition, antioxidant supplementation with riboflavin (vitamin B_2_) significantly decreased inflammatory markers, while it increased systemic levels of free thiols in patients with Crohn's disease, demonstrating that antioxidant therapy holds promise in diseases which are characterized by overproduction of reactive species.^44^

A recent meta‐analysis study reported a significant positive association between NAFLD and all‐cause mortality.^46^ Thus, there is importance for an early and non‐invasive screening method to enable prediction for all‐cause mortality in NAFLD.^47^ Of note, measuring free thiols in serum is relatively minimally invasive. In this study, using Cox proportional hazard regression analysis, we showed a significant predictive association between protein‐adjusted serum free thiols and the risk of all‐cause mortality for subjects with FLI ≥ 60. This association lost its significance after adjustment for potential confounders in subjects with a FLI < 60 and remained significant in subjects with a FLI ≥ 60 (Table 4). Since serum free thiols could be a potential therapeutic target in NAFLD, interventions targeted to increase the free thiol pool could also potentially predict the risk of all‐cause mortality. Taken together, protein‐adjusted serum free thiols could be a prominent predictor of all‐cause mortality in NAFLD. However, it is important to further investigate the association between serum free thiol levels and different stages of NAFLD.

Our study has several strengths and limitations that need to be acknowledged. For example, to the best of our knowledge, this is the first large study to report a significant association between serum free thiols ‐ as a minimally invasive method to quantify systemic oxidative stress ‐ and NAFLD. Most importantly, the protein‐adjusted serum free thiol level was significantly associated with the risk of all‐cause mortality in patients with identified NAFLD. We were able to establish this association in a population‐based cohort study with a large sample size (n = 5562) that enabled us to properly adjust for potential confounding variables with sufficient study power. Furthermore, the association of serum free thiols with suspected NAFLD individuals in the general population were determined using two different, but accurate proxies of NAFLD: the FLI and HSI indices. However, FLI cannot identify absolute clinical NAFLD because of the lack of discrimination between severe steatosis levels and liver fat, but it is considered to be an acceptable method to indicate NAFLD in large‐population based studies.^48^ Although the HSI has only been validated in Asian populations, results were comparable in our cohort. Indeed, both methods are widely accepted and recommended to characterize NAFLD in large population‐based cohort studies.^4^, ^5^, ^26^ However, several study limitations need to be addressed as well. For instance, the PREVEND cohort study mainly comprises individuals of European descent, which are predominantly derived from Caucasian populations, limiting the external applicability of our results to other ethnic populations. In addition, in the PREVEND cohort, it was not feasible to determine NAFLD by other diagnostic methods like liver ultrasound or liver biopsy. Lastly, the association between redox homeostasis and the severity of NAFLD might be important.^49^ However, it was not possible to correlate free thiols with the different stages of NAFLD, e.g. NASH, fibrosis or cirrhosis because of the lack of necessary data to enable this characterization. Similarly, it was not possible to exclude other potential causes of liver disease as these data were not available in the present cohort.

In conclusion, protein‐adjusted serum free thiol concentrations were significantly reduced in subjects with suspected NAFLD, even after adjustment for known risk factors for NAFLD. Furthermore, protein‐adjusted serum free thiols were significantly associated with the risk of all‐cause mortality in subjects with suspected NAFLD. Future studies are warranted that focus on the clinical utility of systemic free thiols in patients with NAFLD and the detailed discovery of potential associations with therapeutic outcome, disease course and overall prognosis. As free thiols are known to be receptive for therapeutic manipulation, future thiol‐targeted therapy should be investigated as well to ameliorate disease outcome in NAFLD.

## CONFLICT OF INTEREST

The authors declare no conflict of interest.

## Supporting information

Supplementary Material
