# Supplementary material for: Oxidative stress is associated with suspected non‐alcoholic fatty liver disease and all‐cause mortality in the general population
Source: Liver Int. 2020 Jun 28;40(9):2148–59. doi: 10.1111/liv.14562 (PMC7496868; doi:10.1111/liv.14562)
Supplement: Supplementary file 1 — Supplementary Material [file LIV-40-2148-s001.docx]

**Oxidative Stress Is Associated with Suspected Non-Alcoholic Fatty Liver Disease and All-Cause Mortality in the General Population**

Turtushikh Damba^1*^, Arno R. Bourgonje^1*^, Amaal E. Abdulle^2^, Andreas Pasch^3^, Svenja Sydor^4^, Eline H. van den Berg^1^, Ron T. Gansevoort^5^, Stephan J.L. Bakker^5^, Hans Blokzijl^1^, Robin P.F. Dullaart^6^, Harry van Goor^7#^, Han Moshage^1,8#^

**Supplemental tables**

|  | Model 1 | | | Model 2 | | Model 3 | | Model 4 | |
| --- | --- | --- | --- | --- | --- | --- | --- | --- | --- |
|  | **OR [95% CI]** | | ***P*-value** | **OR [95% CI]** | ***P*-value** | **OR [95% CI]** | ***P*-value** | **OR [95% CI]** | ***P*-value** |
| Free thiols (2log) | 0.62 [0.51-0.75] | | <0.001 | 0.71 [0.59-0.87] | 0.001 | 0.77 [0.62-0.95] | 0.01 | 0.87 [0.68-1.10] | 0.24 |
| Age |  |  | | 1.01 [1.01-1.02] | <0.001 | 0.99 [0.98-0.99] | <0.001 | 0.98 [0.98-0.99] | <0.001 |
| Sex (reference= male) |  |  | | 1.08 [0.95-1.21] | 0.24 | 1.20 [1.05-1.37] | 0.008 | 1.20 [1.03-1.39] | 0.02 |
| Diabetes (no=reference) |  |  | |  |  | 5.06 [3.43-7.45] | <0.001 | 4.82 [2.88-8.06] | <0.001 |
| Current smoking (reference= no) |  |  | |  |  | 0.68 [0.59-0.79] | <0.001 | 0.67 [0.57-0.79] | 0.001 |
| Use of alcohol (reference= no) |  |  | |  |  | 0.64 [0.56-0.74] | <0.001 | 0.67 [0.57-0.78] | <0.001 |
| Systolic blood pressure |  |  | |  |  | 1.03 [1.02-1.03] | <0.001 | 1.03 [1.02-1.03] | <0.001 |
| Total cholesterol |  |  | |  |  | 1.20 [1.13-1.28] | <0.001 | 1.19 [1.11-1.28] | <0.001 |
| hs-CRP |  |  | |  |  |  |  | 1.04 [1.03-1.06] | <0.001 |

**Supplemental table 1**: Multivariable logistic regression analysis to test the relationship between HSI and levels of protein-adjusted serum free thiols (^2^log-transformed).

Model 1: crude

Model 2: model 1 + additional correction for age and sex

Model 3: model 2 + additional correction for systolic blood pressure, diabetes, current smoking, use of alcohol and total cholesterol

Model 4: model 3 + additional correction for hs-CRP

| Variable | Total (*n*) | OR^*^ | 95% CI | *P*_interaction_  (interaction) |  |
| --- | --- | --- | --- | --- | --- |
| Overall | 5562 | 0.77 | 0.62-0.95 | **0.010** |  |
| Sex |  |  |  |  |  |
| Female | 2815 | 0.74 | 0.56-0.99 | **0.040** |  |
| Male | 2639 | 0.82 | 0.60-1.12 |  |  |
|  |  |  |  |  |  |
| BMI |  |  |  |  |  |
| < 25.0 | 2139 | 1.29 | 0.35-4.76 | 0.378 |  |
| > 25.0 | 3275 | 0.87 | 0.68-1.11 |  |  |
|  |  |  |  |  |  |
| Albuminuria |  |  |  |  |  |
| No | 4813 | 0.75 | 0.59-0.94 | 0.926 |  |
| Yes | 639 | 0.96 | 0.57-1.63 |  |  |
|  |  |  |  |  |  |
| Hypertension |  |  |  |  |  |
| No | 3765 | 0.79 | 0.61-1.04 | **0.028** |  |
| Yes | 1690 | 0.74 | 0.54-1.03 |  |  |
|  |  |  |  |  |  |
| CVD history |  |  |  |  |  |
| No | 5273 | 0.79 | 0.64-0.98 | 0.211 |  |
| Yes | 181 | 0.29 | 0.09-0.94 |  |  |
|  |  |  |  |  |  |
| Diabetes |  |  |  |  |  |
| No | 5322 | 0.76 | 0.61-0.94 | 0.423 |  |
| Yes | 132 | 1.01 | 0.33-3.09 |  |  |
|  |  |  |  |  |  |
| Smoking |  |  |  |  |  |
| No | 3936 | 0.81 | 0.64-1.04 | 0.726 |  |
| Yes | 1518 | 0.66 | 0.44-0.99 |  |  |
|  |  |  |  |  |  |
| Alcohol consumption |  |  |  |  |  |
| No | 1336 | 0.80 | 0.55-1.18 | 0.534 |  |
| Yes | 4118 | 0.76 | 0.59-0.97 |  |  |
|  |  |  |  |  |  |
| Hypercholesterolemia |  |  |  |  |  |
| No | 3904 | 0.69 | 0.54-0.89 | **0.002** |  |
| Yes | 1579 | 0.96 | 0.66-1.39 |  |  |
|  |  |  |  |  |  |

**Supplemental table 2:** Stratified analyses for the association between ^2^log-transformed protein-adjusted serum free thiols and the hepatic steatosis index (HSI) across various subgroups. Stratifications by sex, hypertension and hypercholesterolemia showed significant interactions. ^*^Adjusted for potential confounding factors (sex, age, history of diabetes, current smoking, alcohol consumption, blood pressure and hypercholesterolemia). Abbreviations: CV, cardiovascular; OR, odds ratio; CI, confidence interval; BMI, body-mass index; CVD, cardiovascular disease.

| **A: HSI <36** | | | | |
| --- | --- | --- | --- | --- |
|  |  | Tertiles of protein-adjusted serum free thiols | | |
|  | HR per doubling | < 4.65 μmol/g | 4.65 – 5.46 μmol/g | > 5.46 μmol/g |
| Model 1 | 0.31 [0.22-0.45]  ***P*<0.001** | 1.00 (Reference) | 0.46 [0.34-0.64]  ***P*<0.001** | 0.35 [0.25-0.50]  ***P*<0.001** |
| Model 2 | 0.73 [0.47-1.12]  *P*=0.15 | 1.00 (Reference) | 0.74 [0.54-1.03]  *P*=0.07 | 0.78 [0.54-1.12]  *P*=0.17 |
| Model 3 | 0.76 [0.49-1.18]  *P*=0.22 | 1.00 (Reference) | 0.77 [0.56-1.08]  *P*=0.12 | 0.78 [0.54-1.13]  *P*=0.20 |
| Model 4 | 0.72 [0.44-1.19]  *P*=0.20 | 1.00 (Reference) | 0.86 [0.61-1.22]  *P*=0.39 | 0.75 [0.50-1.13]  *P*=0.17 |
| **B: HSI>36** | | | | |
|  |  | Tertiles of protein- adjusted serum free thiols | | |
|  | HR per doubling | < 4.65 μmol/g | 4.65 – 5.46 μmol/g | > 5.46 μmol/g |
| Model 1 | 0.24 [0.13-0.45]  ***P*<0.001** | 1.00 (Reference) | 0.47 [0.27-0.79]  ***P*=0.005** | 0.25 [0.12-0.52]  ***P*<0.001** |
| Model 2 | 0.54 [0.27-1.08]  *P*=0.08 | 1.00 (Reference) | 0.79 [0.46-1.35]  *P*=0.39 | 0.46 [0.22-0.96]  ***P*=0.04** |
| Model 3 | 0.53 [0.26-1.06]  *P*=0.07 | 1.00 (Reference) | 0.78 [0.45-1.34]  *P*=0.36 | 0.46 [0.22-0.96]  ***P*=0.04** |
| Model 4 | 0.51 [0.23-1.12]  *P*=0.09 | 1.00 (Reference) | 0.80 [0.45-1.43]  *P*=0.45 | 0.39 [0.16-0.94]  ***P*=0.04** |

**Supplemental table 3:** Cox proportional hazards regression models of the association between (^2^log-transformed) protein-adjusted serum free thiols and potential confounding factors with and all-cause mortality, for patients with HSI<36 (A) and HSI>36 (B). Model 1: crude. Model 2: model 1, age- and sex-adjusted. Model 3: model 2, adjusted for systolic blood pressure, diabetes, current smoking, use of alcohol, and total cholesterol. Model 4: model 3, additionally adjusted for hs-CRP. **Bold** *P*-values indicate statistical significance. Abbreviations: HR, hazard ratio.
